# Balanced translocation linked to psychiatric disorder, glutamate, and cortical structure/function

**DOI:** 10.1038/npjschz.2016.24

**Published:** 2016-08-10

**Authors:** Pippa A Thomson, Barbara Duff, Douglas H R Blackwood, Liana Romaniuk, Andrew Watson, Heather C Whalley, Xiang Li, Maria R Dauvermann, T William J Moorhead, Catherine Bois, Niamh M Ryan, Holly Redpath, Lynsey Hall, Stewart W Morris, Edwin J R van Beek, Neil Roberts, David J Porteous, David St. Clair, Brandon Whitcher, John Dunlop, Nicholas J Brandon, Zoë A Hughes, Jeremy Hall, Andrew McIntosh, Stephen M Lawrie

**Affiliations:** 1 Medical Genetics Section, Centre for Genomic and Experimental Medicine, University of Edinburgh, MRC Institute of Genetics and Molecular Medicine at the University of Edinburgh, Western General Hospital, Edinburgh, UK; 2 Division of Psychiatry, Deanery of Clinical Sciences, University of Edinburgh, Royal Edinburgh Hospital, Morningside Park, Edinburgh, UK; 3 Clinical Research Imaging Centre (CRIC), The Queen’s Medical Research Institute, University of Edinburgh, UK; 4 McGovern Institute for Brain Research, Massachusetts Institute of Technology, Cambridge, MA, USA; 5 Institute of Medical Sciences, University of Aberdeen, Aberdeen, UK; 6 Clinical & Translational Imaging Group, Pfizer Global Research, Cambridge, MA, USA; 7 Neuroscience Research Unit, Pfizer Global Research, Cambridge, MA, USA; 8 AstraZeneca Neuroscience, Innovative Medicines and Early Development Biotech Unit, AstraZeneca, Cambridge, MA, USA; 9 Neuroscience and Mental Health Research Institute, Cardiff University, Hadyn Ellis Building, Cardiff, UK

## Abstract

Rare genetic variants of large effect can help elucidate the pathophysiology of brain disorders. Here we expand the clinical and genetic analyses of a family with a (1;11)(q42;q14.3) translocation multiply affected by major psychiatric illness and test the effect of the translocation on the structure and function of prefrontal, and temporal brain regions. The translocation showed significant linkage (LOD score 6.1) with a clinical phenotype that included schizophrenia, schizoaffective disorder, bipolar disorder, and recurrent major depressive disorder. Translocation carriers showed reduced cortical thickness in the left temporal lobe, which correlated with general psychopathology and positive psychotic symptom severity. They showed reduced gyrification in prefrontal cortex, which correlated with general psychopathology severity. Translocation carriers also showed significantly increased activation in the caudate nucleus on increasing verbal working memory load, as well as statistically significant reductions in the right dorsolateral prefrontal cortex glutamate concentrations. These findings confirm that the t(1;11) translocation is associated with a significantly increased risk of major psychiatric disorder and suggest a general vulnerability to psychopathology through altered cortical structure and function, and decreased glutamate levels.

## Introduction

A balanced t(1;11) translocation was first described as a single locus major risk factor for major psychiatric disorder, including schizophrenia, bipolar disorder, and recurrent major depression, in a multiply affected Scottish pedigree with a maximum parametric LOD score of 7.1.^1,2^ The translocation breakpoint lies within the *Disrupted in schizophrenia 1* (*DISC1*) and DISC1FP1/Boymaw genes.^3^ *DISC1* encodes a multi-functional scaffold protein that mediates several processes that have been implicated in the etiology of major psychiatric disorders. Studies utilizing a variety of rodent models have also shown DISC1 to influence neurodevelopment, brain function, and behaviors thought to model schizophrenia and depression.^4,5^ DISC1 has been shown to mediate: neuronal proliferation, differentiation, migration, and integration, neuronal signaling and synaptic plasticity,^4–6^ regulation of neurogenesis and dendritic arborization, and the integration of cortical neurons.^7–10^ In addition, the expression of DISC1, DISC1FP/Boymaw, and the fusion protein caused by the translocation results in severe mitochondrial dysfunction.^11–14^ So far, there is no consistent evidence for a role for genetic variants in DISC1 in schizophrenia risk, including common variants in *DISC1*,^15^ and the potential role of DISC1 and 25 further candidate genes has been recently challenged.^16,17^ However, findings from the original Scottish t(1;11) family have been presented that support the ‘common disease; rare variant’ hypothesis and suggest that DISC1 may have a role in major psychiatric disorders.^18^ The present study updates the linkage evidence for the t(1;11) translocation and risk of psychiatric disease and reports new neuroimaging measures from the family that provide evidence for biological consequences of the translocation.

We have previously reported that the P300 event-related potential measure of attention is consistently altered among t(1;11) carriers in a manner comparable to that seen in schizophrenia.^2^ Until now, we have not had the opportunity to test for the possible impact of the t(1;11) translocation on measures of brain structure, function, and metabolite concentrations. Several cognitive and clinical neuroimaging measures are highly heritable including measures of cortical thickness (CT), fractional anisotropy, and brain activation measures.^19–21^ The structure and function of the prefrontal cortex are implicated in a range of psychiatric disorders (such as schizophrenia, attention-deficit/hyperactivity disorder, and autism); they have high levels of heritability and are commonly reported to be abnormal in unaffected relatives of patients.^22^ Studies examining the effects of common variants in DISC1 alleles in humans, although inconsistent, have suggested that variation at the DISC1 locus contributes to structural and functional changes across the brain, but particularly in prefrontal and temporal regions.^5,23–25^ DISC1 has also been shown to regulate the healthy functioning of *N*-methyl-d-aspartate receptors (NMDARs).^26–28^ A transgenic mouse model expressing a truncated form of Disc1, reflecting the effect of the translocation on the Disc1 protein, shows reduced cortical–hippocampal connectivity, reduced CT, and dysfunction of the glutamate system including reduced expression of NMDAR subunits in the hippocampus.^10,29^

Recent studies have suggested that the genetic risk associated with alterations in glutamatergic function may be implicated in the pathophysiological pathways of major psychiatric disorders. The glutamate hypothesis of schizophrenia is based on the NMDAR hypofunction model.^30^ Importantly, the glutamate hypothesis encompasses, rather than negates, the dopamine hypothesis of schizophrenia^31–33^ and is a possible final common interaction pathway for genetic risk factors for schizophrenia.^34^ The *N*-methyl-d-aspartate subtype of the glutamate receptor is implicated across anatomical, cellular, neurochemical, and neuronal levels in the development of schizophrenia,^35^ and the glutamate hypothesis provides arguably the best available account of the positive, negative, and cognitive symptoms seen in schizophrenia. Glutamatergic disruption has also been implicated in bipolar disorder and major depression.^36,37^ Furthermore, it is increasingly clear that major psychiatric disorders such as schizophrenia, bipolar disorder, and major depression share at least some genetic risk factors.^38^

Convergent findings supporting glutamatergic models have been reported from preclinical and clinical studies of the role of NMDARs during working memory (WM) coping and WM impairment after NMDAR antagonist treatment.^39–43^ Functional magnetic resonance imaging (fMRI) studies in humans have presented evidence for ketamine-induced effects on both WM and prefrontal region activation.^39,40^ The role of dorsolateral prefrontal cortex (DLPFC) dysfunction in WM deficits has been related to dopaminergic alterations in schizophrenia, bipolar disorder, and depression, but also to glutamatergic alterations and more specifically to dopamine–glutamate interactions.^31,42,44^ Magnetic resonance spectroscopy (MRS) provides a means to measure glutamate concentrations in circumscribed regions *in vivo* in humans. MRS studies have demonstrated alterations of glutamatergic concentrations in prefrontal regions in bipolar disorder and depression as well as schizophrenia.^37^

The primary aims of this study were to re-visit the multiply affected family segregating the t(1;11) translocation, generate non-parametric LOD scores across diagnoses on the updated pedigree and to investigate the effect of the t(1;11) translocation on brain structure, brain metabolite concentrations and brain function by imaging of family members with and without the translocation.

## Results

### Extension of the family increases the evidence of linkage between the translocation and major mental illness

We previously published significant linkage of the t(1;11) translocation to major mental illness in a single Scottish family.^2^ This translocation is a unique variant private to this family. We therefore sought to confirm this linkage through the recruitment of additional members of the family to the study and full clinical re-evaluation of all participants by two psychiatrists. Clinical re-evaluation using all available current and historic information was undertaken on the full family as reported in Blackwood *et al.*^2^ Forty-two participants who took part in the previous studies volunteered for the present study. An additional 25 participants took part for the first time including four who carry the translocation. Historical information was reviewed for the remaining individuals. The extended family comprised 107 individuals in six generations and translocation status was determined in all but one individual (for whom no DNA was available and translocation status could not be imputed). In total, 37 participants carried the t(1;11) translocation and 69 were non-carriers. Details of diagnoses are given in Table 1. Two-point variance component analyses were performed using the program SOLAR (Table 2). To facilitate the comparison of these results with those of the t(1;11) family as reported in Blackwood *et al.*,^2^ variance component LOD scores were also generated on the previously published pedigree. In the extended family, the t(1;11) translocation was significantly linked to a phenotype that includes: only schizophrenia and schizoaffective disorder (LOD=3.3); only affective disorders (bipolar disorder and recurrent major depression) (LOD=3.5); or all cases of major mental illness (schizophrenia, schizoaffective disorder, bipolar disorder, and recurrent major depressive disorder; LOD=6.1). A maximum LOD score was obtained if the phenotype is further extended to include three cases with cyclothymia (LOD=7.9).

### t(1;11) translocation carriers show localized differences in CT and gyrification index

Brain structural abnormalities, that have been identified between individuals with schizophrenia and unaffected individuals, are associated with both large genomic rearrangements and single-nucleotide polymorphisms including those in *DISC1*.^45–50^ We sought to identify the effects of the translocation on CT and cortical folding. Useable structural MRI, data were acquired for 12 t(1;11) translocation carriers and 18 non-carriers (Table 1). The average CT±s.d. was 2.40±0.16 mm for non-carriers and 2.23±0.12 mm for translocation carriers. The difference in CT between the two groups was statistically significant (F(1,26)=4.248, *P=*0.049), with greater CT in non-carriers compared with carriers. The average local gyrification index (LGI)±s.d.: 2.94±0.19 for non-carriers and 2.78±0.13 for carriers. The one-way analysis of covariance, covarying for age and sex, found statistically significant differences of LGI between groups (F(1,28)=6.558, *P=*0.016). These results were, however, not robust after controlling for reduced intra-familial relatedness (global CT *P=*0.055 and LGI *P=*0.23). No significant differences were found in the global cortex surface area or estimated intracranial volume (*P*>0.05).

**Figure 1:**
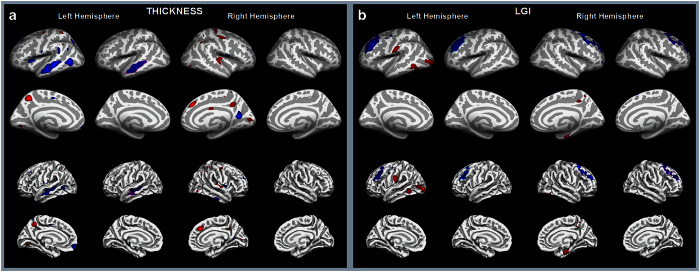
Effect of the translocation on cortical thickness and local gyrification index. (**a**) Cortical thickness and (**b**) local gyrification difference between translocation carriers and non-carriers rendered on the inflated and non-inflated cortical surface of the left and right brain templates. Columns 1, 3, 5, and 7 show the significance map of the difference; columns 2, 4, 6, and 8 show the regions that survive the cluster-wise multiple comparisons correction (*P*<0.05). The blue color indicates that the cortex is thinner and less gyrified for the translocation carriers compared with non-carriers, whereas the red color indicates the opposite effect. All these analyses are controlled for age and sex. Note however that only left superior temporal sulcus cortical thickness and right superior frontal sulcus gyrification index are robust to controlling for intra-familial relatedness (see text). LGI, local gyrification index.

Localized differences, many bilateral, in CT and LGI were found between t(1;11) translocation carriers and non-carriers on controlling for age and sex, and multiple comparisons (Figure 1), but only reduced left superior temporal sulcus (STS) CT in the temporal lobe and reduced right superior frontal sulcus LGI in the DLPFC were robust to controlling for intra-familial relatedness (*P=*0.022 and *P=*0.025, respectively).

All subgroups of t(1;11) translocation carriers had similarly low left STS CT and right superior frontal LGI values (Supplementary Results). LGI results for these regions split by translocation carrier diagnosis (psychosis, recurrent depression, and other) are shown in Supplementary Figure 1.

We examined the association between these structural measures and the contemporaneous mental state assessments with the Positive and Negative symptom scale (PANSS) in 30 subjects (both carriers and non-carriers). PANSS general psychopathology scores were negatively correlated with the left STS CT (*r*=−0.43, *P=*0.016) and also with right superior frontal LGI (*r*=−0.41 (*P=*0.025). PANSS-positive scores were negatively correlated with left STS CT (*r*=−0.36, *P=*0.048), but not with the right superior frontal LGI (*r*=−0.22, *P=*0.23). This suggests that the structural deficits in these regions effect symptomology, possibly through an impact on social cognition.

### t(1;11) translocation carriers show increased activation in the caudate nucleus on increasing verbal WM load

Analyses of fMRI blood oxygen level-dependent activation profiles enable the *in vivo* study of brain activity during specific cognitive tasks. Patients with schizophrenia have been shown to have overactivation of brain regions during WM tasks,^51^ although reduced activation in contrast to healthy controls has also been reported.^52^ We sought evidence of an effect of the translocation on brain activation with increasing WM load. Useable fMRI data were acquired for eight family members with the t(1;11) carriers and 15 non-carriers (Table 1). These data were collected after the structural MRI and MRS acquisitions. Useable fMRI data were acquired for eight family members with the t(1;11) carriers and 15 non-carriers (Table 1).

Reaction time in the verbal WM ‘N-back’ task showed a trend for a main effect of WM load (F(2,36)=2.571, *P=*0.09), but no significant effect of the group (F(1,18)=0.018, *P=*0.89), or the group×WM load interaction (F(2,36)=1.537, *P=*0.23). The sensitivity index (*d’*) was computed to assess the behavioral performance between the groups. We found neither a significant main effect of WM load (F(2,36)=1.24, *P=*0.30) nor group (F(1,18)=0.930, *P=*0.35) Furthermore, the group×WM load interaction was not significant (F(2,36)=0.22, *P=*0.80).

**Figure 2:**
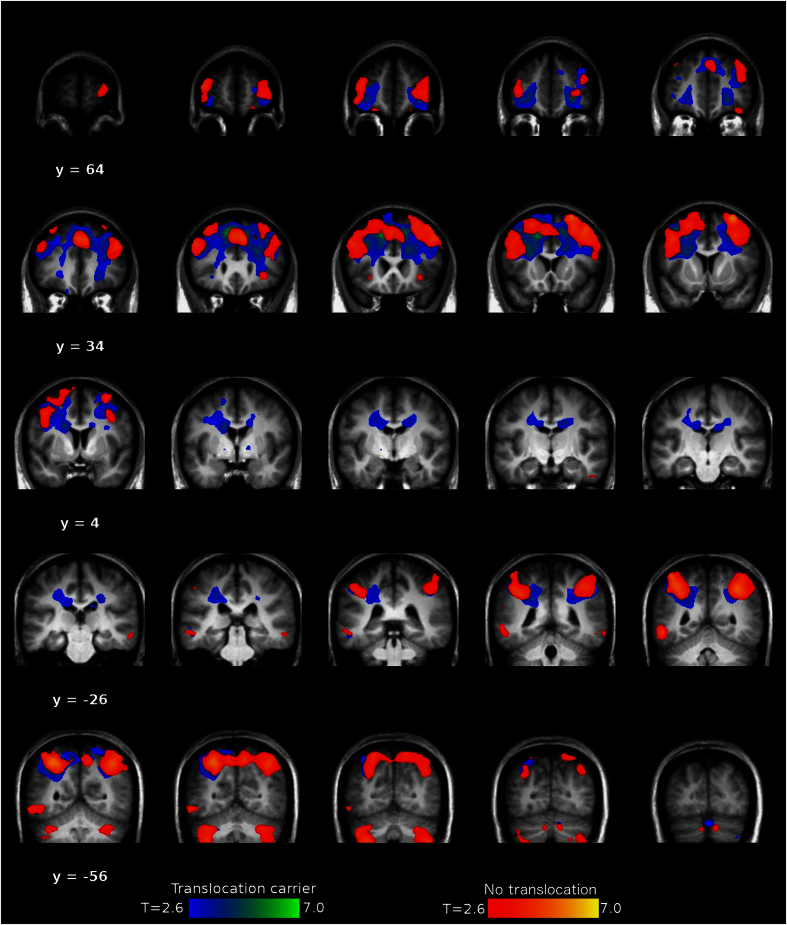
Effect of increasing working memory load on blood oxygen level-dependent activity measures in translocation carriers and non-carriers. Coronal sections through the brain to show the effects of increasing working memory load (from 0- to 1- to 2-back) in the N-back task on functional MRI in t(1;11) translocation carriers and non-carriers. The image is thresholded at *P*<0.001, uncorrected, to show regional activations. These were statistically significant in/across both groups in bilateral inferior, middle, and superior frontal cortices, bilateral inferior parietal lobules, right cerebellum, left inferior temporal gyrus, and the left middle orbital gyrus at *P*<0.05, family-wise error corrected for multiple comparisons. There were no statistically significant group differences at a family-wise error-corrected threshold of *P*<0.05.

**Figure 3:**
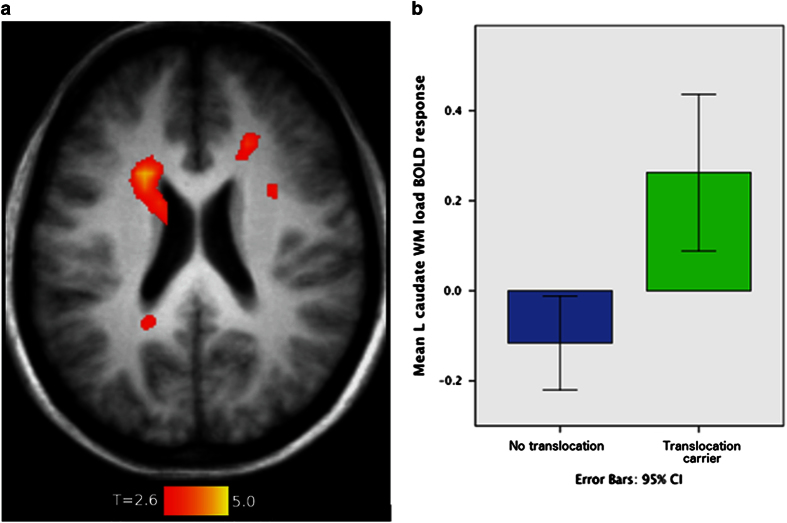
Differential brain activation during increasing working memory load between translocation carriers and non-carriers. The effects of increasing working memory load (from 0- to 1- to 2-back) in the N-back task on functional MRI comparing t(1;11) translocation carriers and non-carriers, controlling for age and sex. (**a**) Transverse slice (*z*=22) displaying the statistically significant group×load interaction in left caudate, a family-wise error-corrected *P*<0.05. (**b**) Contrast estimates in the left caudate for 2-back>0-back for t(1;11) carriers and non-carriers to indicate the size of the effect.

When evaluating the effect of increasing WM load on blood oxygen level-dependent activation across translocation carriers and non-carriers, significant activation was found in bilateral inferior, middle and superior frontal cortices, bilateral inferior parietal lobules, right cerebellum, left inferior temporal gyrus, and the left middle orbital gyrus (*P*<0.05, Figure 2). No regions demonstrated statistically significant group differences (*P*>0.05). There was a significant group×WM load interaction in left caudate nucleus, with greater activation in translocation carriers with increasing load from 0-, 1-, to 2-back (Figure 3; F(1,17.5)=24.95, *P*<0.001), which was robust to controlling for familial relatedness (*P=*0.001). Within the translocation carriers, caudate WM load response did not correlate with PANSS symptom measures (*P*>0.05). The overactivation of the left caudate nucleus in t(1:11) translocation carriers may indicate inefficient cortico-striatal information processing which is important in movement control, mood, and higher cognitive function.

### t(1;11) translocation carriers show reduced levels of glutamate in the right dorsolateral prefrontal cortex

Metabolites reflecting glutamate neurotransmission have been shown, in some studies, to differ between patients and controls.^31,42^ We investigated the effect of the translocation on glutamate and *N*-acetylaspartate (NAA) concentrations in prefrontal regions using MRS. Levels of glutamate and NAA were measured in the bilateral DLPFC and anterior cingulate cortex (ACC) of translocation carriers and non-carriers. The results are reported separately for each region.

In the right DLPFC, translocation carriers had significantly lower levels of glutamate than non-carriers (*N*=12 carriers vs. *N*=16 non-carriers, mean glutamate levels±s.d.: 7.72±1.6 vs. 9.86±2.4 mmol/l respectively, *P=*0.021). The diagnosis subgroups within translocation carriers had similarly low levels of glutamate (psychosis (*N*=3) 6.9 mmol/l, recurrent MDD (*N*=3) 7.4 mmol/l, and others (*N*=6) 7.7 mmol/l). Correlations between glutamate levels and PANSS general psychopathology and positive symptom scales were non-significant (*P*>0.4). In the left DLPFC, there was no significant effect of carrying the translocation on glutamate levels (7.7±1.1 vs. 8.3±1.7 mmol/l, respectively, *P=*0.42). Left DLPFC NAA levels were significantly lower in the translocation carriers (*N*=11 carriers vs. *N*=16 non-carriers, mean NAA levels±s.d. was 11.9±2.1 and 13.7±1.8 mmol/l, respectively, *P=*0.025), although these were not robust to controlling for reduced intra-familial relatedness. NAA levels were not significantly lower in the right DLPFC (*N*=12 carriers vs. *N*=16 non-carriers, mean NAA levels±s.d. was 13.1±2.5 and 14.3±1.6 mmol/l, respectively, *P=*0.17).

There were no significant differences between translocation carriers and non-carriers in the concentrations of glutamate or NAA in the ACC (*P*>0.05).

These data suggest a specific glutamate deficit in the DLPFC of t(1;11) translocation carriers, consistent with a direct genetic effect.

## Discussion

The current study provides a contemporary and extensive follow-up to earlier work on the t(1;11) translocation and confirms that it is a rare variant of large effect, linked with genome-wide significance to a broad psychiatric phenotype that includes schizophrenia, bipolar disorder, and recurrent major depression and, shown here for the first time, is associated with structural and functional changes to the brain detected by neuroimaging.

Specifically, carriers of the t(1;11) translocation demonstrate reductions in glutamate concentrations in the right DLPFC consistent with the glutamate hypothesis, as well as reduced CT and gyral folding, and increased left caudate nucleus blood oxygen level-dependent activation when compared with non-carriers. Of these, only the localized CT and gyrification reductions were associated with general psychopathology and only the left STS CT reductions were associated with positive symptom severity in t(1;11) translocation carriers. Elsewhere, we have shown widespread reductions in white matter tract integrity in t(1;11) translocation carriers.^53^ These striking effects were observed in what are relatively small comparative studies that benefit from being part of a long-term within-family study of the incidence and evolution of psychiatric illness, controlled for by the presence or absence of the t(1;11) translocation. The within-family design optimizes the genetic matching over the genome. However, the correspondingly low numbers may have limited our ability to detect some effects of the translocation, and correlations between these effects and symptom correlations, but did not obscure the impact of the t(1;11) translocation on aspects of human brain structure, function, and neurochemistry.

The t(1;11) translocation was strongly associated with a range of psychiatric outcomes in the family, but with more homogeneous effects on imaging measures. This pattern of results, of a greater genetic impact on neurobiological measures than clinical phenotypes, is consistent with the previous P300 event-related potential study of the t(1;11) family, which found abnormalities in those with the translocation regardless of clinical diagnosis.^2^ Comparable effects of major psychiatric illness on human brain imaging measures,^54–56^ as well as a reduction in DLPFC glutamate concentration,^37^ have been noted in multiple case–control studies, transcending conventional diagnostic boundaries. The pattern of neuroimaging abnormalities detected in this study is striking and reflective of the pattern identified in a mouse model of the translocation in which a truncated *Disc1* fragment is expressed in a single copy (*Disc1tr* hemizygous, Hemi, mice).^10^ Similarly to the family, this mouse model shows both cortical thinning and deficits in the glutamate system,^10,29^ suggesting that these deficits, at least in part, may be the direct result of disruption of the *DISC1* locus.

We found relatively few correlations between the neuroimaging measures and symptom severity in participants, nor any apparent instances where the effects of the t(1;11) translocation could be accounted for by extreme results in one or more diagnostic subgroups (Supplementary Data). This supports the interpretation that the brain imaging abnormalities evident in the t(1;11) carriers are primarily genetic in origin and confer risk across a range of phenotypes. The abnormalities in CT are in keeping with the generalized reductions in CT reported in many psychiatric disorders, e.g., Hulshoff Pol *et al.*^57^ Additional structural genetic variants, polygenic risk factor loads, and environmental risk factors may then mediate the development of schizophrenia, bipolar disorder, and recurrent depression. In this respect, longitudinal, within-family studies can be a powerful complement to large case–control studies for which clinical heterogeneity may obscure underlying commonalities.

In conclusion, our results substantiate prior evidence for a genome-wide significant effect of the t(1;11) translocation on cross-disorder risk of major mental illness. The idea that study of rare genetic variants can highlight biological mechanisms of relevance is not new, indeed is widely accepted and acknowledged as providing valuable insights, as, e.g., the single-gene risk factors for dementia (APP, PSEN1, and 2), and the role of copy-number variants and *de novo* mutations of high penetrance in schizophrenia and autism spectrum disorder.^58–60^ The t(1;11) translocation, similarly, provides a clear-cut and useful biological model for major psychiatric disorder. The most parsimonious explanation is that the molecular mechanism is explained by disruption of genes on chromosomes 1 and 11, including the *DISC1* gene; a mechanism supported by the wealth of evidence linking DISC1 biology to independently constructed core concepts in psychopathology. As such, our findings suggest translational opportunities for experimental studies on carefully phenotyped and genetically analyzed subjects that may generalize to the wider population to speed the discovery and evaluation of much needed evidence based interventions.

## Materials and methods

### Participants

Individuals with and without the t(1;11) translocation were recruited from a previously reported extended Scottish family.^1,2,61^ Some of the family had been in contact with members of the research team for many years and through them other members of the family were invited to participate. None of the family members who participated suffered from substance dependence or neurological injury or illness or had MRI safety preclusions. A summary of the number of individual participants in each study, their translocation status, and medication at the time of each study is given in Table 1.

### Clinical and cognitive assessment

Psychiatric diagnosis according to DSM-IV (TR) criteria was established by consensus between two trained psychiatrists (D.B. and A.W., one of whom (A.W.) was blind to the individuals karyotype status). Diagnostic information was obtained by a face-to-face semi-structured interview using the Structured Clinical Interview for DSM-IV (SCID)^62^ supplemented by reviews of hospital records and collateral information from hospital psychiatrists and general practitioners. At the time of interview, the following ratings were completed: PANSS;^63^ Scale for the Assessment of Negative Symptoms;^64^ Global Assessment of Function;^65^ Young Mania Rating scale;^66^ and the Hamilton Depression Rating Scale.^67^ Current and premorbid IQ were assessed using the National Adult Reading Test^68^ and the Wechsler Abbreviated Scale of Intelligence (WASI).^69^ Historic information reported by StClair *et al.*^1^ and Blackwood *et al.*^2^ was retained and reviewed for subjects who were deceased or not available for follow-up. The operational criteria symptom check-list^70^ was completed based on psychiatric case notes and interview data. Sample demographics are provided in Table 3.

### Genotyping of the translocation

The t(1;11) translocation status of family members was originally ascertained by karyotyping as reported by Jacobs *et al.*,^61^ and subsequently by StClair *et al.*^1^ and Blackwood *et al.*^2^ For the current study, a PCR assay specific for the t(1;11) breakpoint was devised, validated on 22 samples from participants for whom karyotype status was known (including 14 carriers) and used to determine the presence or absence of the t(1;11) in 25 new participants (Supplementary Methods). DNA samples were available for PCR-based verification of translocation status from 48 individuals (18 carriers and 30 non-carriers). Translocation status was imputed in additional family members were possible.

### Multimodal neuroimaging

Neuroimaging measures were: (i) global and local CT, estimated intracranial volume, surface area, and gyrification from index structural MRI, (ii) brain activation during the verbal WM ‘N-back’ task using functional MRI, and (iii) glutamate and NAA levels in the DLPFC and ACC using MRS. Detail of the pre-processing and analysis of the neuroimaging measures are given in Supplementary Methods. Specific details for each imaging modality are included in the results section. Hypothesis testing was undertaken with significance set at *P*<0.05 after correction for multiple comparisons. We further investigated whether any differences between the groups were associated with psychopathology by examining the results for diagnostic subgroups (psychosis, recurrent depression, and others) of those with the translocation, as well as relating imaging measures to the PANSS general and positive psychotic symptom severity ratings in those with and without the t(1;11) translocation.

### Statistics

Two-point variance component linkage analyses of the translocation status with SCID diagnosis were performed using SOLAR software package^71^ under the assumption of the liability threshold model for discrete traits.^72,73^ LOD scores were adjusted for deviation of the phenotype from normality, by correcting the inflation of the observed LOD scores, by the comparison with LOD scores generated using a simulated normally distributed trait with 10,000 permutations, using the lodadj command in SOLAR.^74^ These adjusted LOD scores are presented in the results.

All neuroimaging data group contrasts were conducted controlling for age and sex, and for intra-familial relatedness. Intra-familial relatedness, how related individuals are within the family, was modeled by creating an inverse relationship matrix using pedigree kinship information. Where univariate models of the imaging measures, controlling for age and sex, were nominally significant (*P*<0.05), the analyses were repeated using mixed linear models implemented in ASReml-R (www.vsni.co.uk/software/asreml), fitting the inverse relationship matrix as a random effect, allowing us to control for familial structure. The significance of fixed effects within the model was then assessed using a conditional Wald F-test.

### Study approval

The study was approved by the Multicentre Research Ethics Committee for Scotland (09/MRE00/81). A detailed description of the study was given and written informed consent was obtained from all individuals before participation.

## Figures and Tables

**Table 1:** Study participants

*Participants*	N	*t(1;11)*		*DSM-IV diagnoses (*N*)*		*Medication*	
*Study*		*Y*	*N*	*Carriers*	*Non-carriers*	*Carriers*	*Non-carriers*
Linkage analysis	107^a^	37	69	SCZ (2), SCZAFF (4), BP1 (2), rMDD (8), MDD (4), cyclothymia (3), conduct disorder (3), generalized anxiety (3), no psychiatric disorder (2), inadequate information (6)	rMDD (3), MDD (3), generalized anxiety (1), alcohol dependency (1), no psychiatric disorder (54), inadequate information (7)	NA	NA
Clinical assessment	39	14	25	SCZ (1), SCZAFF (1), BP1 (1), rMDD (3), MDD (3), cyclothymia (3), conduct disorder (1), no psychiatric disorder (1)	rMDD (2), MDD (1), generalized anxiety disorder (1)	Sodium valproate (3), +clozapine (1), +risperidone (1), +sertaline (1)	Amitriptyline (1)
Structural MRI	30	12	18	SCZ (1), schizoaffective (1), BP1 (1) rMDD (3), MDD (2), cyclothymia (3), conduct disorder (1)	rMDD (2), MDD (1)	Sodium valproate (3), +clozapine (1), +risperidone (1), +sertaline (1)	Amitriptyline (1)
MRS	28	12	16	SCZ (1), SCZAFF (1), BP1 (1) rMDD (3), MDD (2), cyclothymia (3), conduct disorder (1)	rMDD (2), MDD (1)	Sodium valproate (3), +clozapine (1), +risperidone (1), +sertaline (1)	Amitriptyline (1)
Functional MRI	23	8	15	rMDD (2), MDD (2), cyclothymia (3), conduct disorder (1)	MDD (1)	None	Amitriptyline (1)

Abbreviations: BP1, bipolar 1; MDD, single episode depression; MRS, magnetic resonance spectroscopy; MRI, magnetic resonance imaging; NA, not available; rMDD, recurrent major depressive disorder; SCZ, schizophrenia, SCZAFF, schizoaffective disorder.

a Translocation status was unavailable for one individual.

**Table 2:** Diagnostic models used for variance component linkage analyses of the original and extended family

*Model*	*Diagnoses*	N			*LOD*
		*t(1;11) carriers*	*t(1;11) non-carriers*	*ALL*	
*Original family*					
Model 1	SCZ, SCZAFF	7	0	7	1.7
Model 2	BP1, rMDD	12	0	12	2.2
Model 3	SCZ, SCZAFF, BP1, rMDD	19	0	19	**3.8**
	Unaffected	7	21	28	
	Unknown	2	1	3	
	Total	30	28	58	
					
*Extended family*					
Model 1	SCZ, SCZAFF	6	0	6	**3.3**
Model 2	BP1, rMDD	10	3	13	**3.5**
Model 3	SCZ, SCZAFF, BP1, rMDD	16	3	19	**6.1**
Model 3+cyclothymia	SCZ, SCZAFF, BP1, rMDD, cyclothymia	19	3	22	**7.9**
	Unaffected	2	53	55	
	Unknown	8	8	16	
	Total	37	70	107	

Abbreviations: BP1, bipolar 1; MDD, single episode depression; rMDD, recurrent major depressive disorder; SCZ, schizophrenia; SCZAFF, schizoaffective disorder.

For comparison, the original family (Blackwood *et al.*^2^) and the extended family two-point LOD scores are shown. Bold=LOD scores>3.

**Table 3:** Sample demographics for the t(1;11) translocation carriers and non-carriers

	*t(1;11) carriers*		*t(1;11) non-carriers*	
Sample size	14		25	
*Gender*				
Male	8		12	
Female	6		13	

	*Mean*	*s.d.*	*Mean*	*s.d.*
Age	55.64	15.28	38.6	20.14
Premorbid IQ^a^	103.07	7.23	104.54	6.36
Current IQ^a^	88.15	16.79	93.47	10.68
*PANSS*				
Total score	46.86	24.38	32.84	4.82
Negative symptoms	9.64	9.06	7	0
Positive symptoms	10.14	6.29	7	0
General Symptoms	27.07	10.73	18.84	4.82

SANS	7.07	26.46	0.24	1.2
GAF	75.14	22.41	88.2	12.82
YMRS	2.93	5.4	NA	NA
HRSD	5.57	6.21	2.8	5.43

Abbreviations: GAF, Global Assessment of Function; HRSD, Hamilton Depression Rating Scale; PANSS, Positive and Negative Symptoms Scale; SANS, Scale for the Assessment of Negative Symptoms; WASI, Wechsler Abbreviated Scale of Intelligence; YMRS, Young Mania Rating Scale.

Premorbid IQ (National Adult Reading Test) and current IQ (WASI).

a *N*=13 in the translocation carriers and *N*=15 in the non-carrier groups.
