# Supplementary material for: Balanced translocation linked to psychiatric disorder, glutamate, and cortical structure/function
Source: NPJ Schizophr. 2016 Aug 10;2:16024–. doi: 10.1038/npjschz.2016.24 (PMC4994153; doi:10.1038/npjschz.2016.24)
Supplement: Supplementary Material [file npjschz201624-s1.doc]

**Balanced translocation linked to psychiatric disorder, glutamate and cortical structure/function**

Full details of the linkage and imaging data are available from the authors to *bone fide* researchers after appropriate Material Transfer Agreements.

**Supplementary Methods**

**PCR typing of translocation breakpoint**

Primers were designed to span the t(1;11) breakpoint (1), using the Primer 3 primer design program (http://bioinfo.ut.ee/primer3/). The translocation primers are as follows:

t(1;11)_chr1 primer TTTCTTTGGAAGGCACCTTATC

t(1;11)_Chr11 primer AGCAAAGTGGGTGAAGAATAGAG

PCR product size 1105bp

DNA was co-amplified, in the same reaction, using DISC1 exon 9 primers to verify the assay function:

DISC1 Ex9 forward primer TTCCCCAGAGGACTGCTAAG

DISC1 Ex9 reverse primer AAATGTCCCCAAGGAAAAGG

PCR product size: 484bp

PCR was performed in total volume of 10 µl with 20 ng DNA, 1 times reaction buffer with 1.5 mM MgCl_2_ (Perkin-Elmer), 100 µM of each dNTP (Peqlab), 0.5 U Taq DNA polymerase (Sigma) and 0.33 µM of each primer (Sigma). PCR cycling was carried out on a PTC-225 thermal cycler (MJ Research). PCR cycling conditions consisted of denaturation at 95°C for 1 min, followed by 10 cycles of 93°C for 20 s, 70°C for 30 s, minus 1°C/cycle and 72°C for 1 min, followed by 30 cycles of 93°C for 20 s, 60°C for 30 s and 72°C for 1 min and a final extension of 72°C for 10 min. Five microlitres of the PCR product was resolved on a 1.5% agarose gel and PCR product size was estimated against 250ng of λ HindIII size standard (Life Technologies).

1. Millar JK, Wilson-Annan JC, Anderson S, Christie S, Taylor MS, Semple CA et al. Disruption of two novel genes by a translocation co-segregating with schizophrenia. Human molecular genetics 2000; 9(9): 1415-1423.

**Structural MRI acquisition and analysis**

Brain scans were collected using a 3 T Siemens Verio (Erlangen, Germany) MRI scanner using a manufacturer-supplied 12-element matrix head coil at the Clinical Research Imaging Centre (CRIC), the Queen’s Medical Research Institute, Edinburgh, UK. After a sagittal localizer, T1-weighted magnetization-prepared rapid-acquisition gradient echo (MPRAGE) MR images were obtained using TR=2300 ms, TE = 2.98 ms, and TI=900 ms, (Flip angle = 9, FOV = 256mm x 256 mm) with an isotropic voxel resolution of 1 mm, parallel to AC-PC plane.

Image processing

To provide good quality anatomical information customized for each subject, the standard FreeSurfer pipeline was modified to incorporate pre-processing steps available in FSL software (http://fsl.fmrib.ox.ac.uk/fsl/fslwiki/): (i) the BET function was used to automatically strip away the skull, (ii) the FAST function was used to correct for intensity variations due to magnetic susceptibility artefacts and radio frequency field inhomogeneities and (iii) the FLIRT function was used to normalise the isolated brain to the MNI152 template brain using a 7 degree of freedom transformation (i.e. 3 translations, 3 rotations and 1 uniform scaling) with preservation of the shape of individual brains. Volume normalisation was achieved by adjustment of the relative position, orientation and size until the mutual information between the individual and the template was maximized. The transformation matrix and the uniform scaling factor were saved for later group analyses.

In the next series of processing steps, the conventional FreeSurfer pipeline (https://surfer.nmr.mgh.harvard.edu/) was applied to the FSL pre-processed brains to reconstruct the high quality brain surfaces: (i) bias field correction was repeated to assist classification of voxel data into different tissue types and to locate the boundaries between different brain compartments, (ii) the white matter was labelled, split into two cerebral hemispheres and the cerebellum and brain stem were removed, (iii) a triangular mesh was fitted to cover the outer voxels of the white matter component for each cerebral hemisphere including its deformation with respect to the intensity gradients between the white and grey matter (yielding a final surface mesh with sub-voxel resolution), (iv) the resulting surface representing the boundary between the cerebral cortex and underlying white matter was expanded along the direction of the intensity gradients between grey matter and CSF until it coincided with the cortex surface, (v) a correction was applied to ensure the surface preserved spherical topology which is necessary to allow the surface to be further inflated to unfold the infolded sulci and subsequently homeomorphically transformed to a sphere space for surface registration purpose (1). The quality of the hemisphere surfaces was visually accessed using the QA tools in FreeSurfer (https://surfer.nmr.mgh.harvard.edu/fswiki/QATools).

For each subject, the cortical thickness at each surface location (or vertex) was defined as the average of the closest distance between the white and the pial surfaces in either direction (2). The area was computed as the average area of the triangles of which the vertex was a member. The volume was computed as a product of the area and the thickness (3). The gyrification index is a metric that quantifies the amount of cortex buried within the sulcal folds as compared with the amount of cortex on the outer visible cortex. This measure was calculated using Freesurfer (as the -localGI flag) which implements a method, based on that of Schaer et al., 2008 (1), which computes local measurements of gyrification at thousands of points over the whole cortical surface. We used a “Local Gyrification Index (LGI)” and an “average Local Gyrification Index (LGI)”. The Local Gyrification Index quantifies the gyrification index in circular three-dimensional regions of interest. The local gyrification (LGI) was computed in the following two-step process, (i) the pial surface was smoothed, (ii) the GI at each vertex was computed as the ratio between the area of a circular Region of Interest (ROI) on smooth surface and the associated ROI on the pial surface (4). For each individual, the thickness, area and volume values at each vertex were scaled by the proper form of the scaling factor (e.g., thickness = thickness/scale, area=area/scale2) that was recorded from the FSL pre-processing to recover to the real world values. There was no need to scale the local gyrification value as it is computed as a ratio.

In addition, the anatomical information (e.g., folding patterns) for each subject was modelled quantitatively by a set of curvature-based descriptors (e.g., the spatial relationship of a vertex to neighbouring vertices) at each surface. The curvature-based descriptors of each hemisphere were used to drive a non-linear registration in spherical space by shifting vertex positions along the sphere surface until a good alignment between the folding pattern of a hemisphere and the patterns of a reference template has been achieved (3,5).

The step of registering hemispheres to the standard template, left and right respective, established inter-subject correspondence between the vertices. This procedure allowed vertex-wise group comparison of the cortical parameters - thickness, gyrification, surface area - after they were re-sampled into standard space. To compensate for stretching or compression, a ‘Jacobian correction’ was included to modulate the area and volume resampling, but it was not necessary for the non-areal measures of cortical thickness and gyrification.

Statistical analyses

Vertex-wise comparison of surface brain parameters

The evaluation of the group difference of the parameters between t(1;11) translocation carrriers and non-carriers was performed at each vertex using the surface-based group analysis tools in FreeSurfer, which is based on the general linear model (GLM). In order to increase the signal-to-noise ratio of the vertex-basis comparison, a Gaussian smoothing kernel with full-width half-maximum of 15 mm was applied to the re-sampled values of parameters. Because of the unbalanced sex and unmatched age, both sex and age were controlled for. To take into account multiple comparisons, a further cluster-wise correction was applied to find the cluster of contiguous vertices with vertex-wise p-value (or cluster-forming threshold) less than 0.05. In brief, a Monte Carlo simulation was run to repeatedly (10000 iterations) 1) synthesize white Gaussian noise on the surface, 2) smoothed, thresholded and clustered to get a measure of the distribution of the maximum cluster size, with which the p-value of a cluster was determined under the null hypothesis (6).

1 Dale, A. M., Fischl, B., Sereno, M.I. Cortical surface-based analysis I: Segmentation and surface reconstruction. NeuroImage 9, 179-194 (1999).

2 Fischl, B. & Dale, A. M. Measuring the thickness of the human cerebral cortex from magnetic resonance images. Proceedings of the National Academy of Sciences 97, 11050-11055 (2000).

3 Greve, D. N. e. a. Surface-based analysis of language lateralization and cortical asymmetry. Journal of Cognitive Neuroscience 25, 1477-1492 (2013).

4 Schaer, M. et al. A surface based approach to quantify local cortical gyrification. IEEE Transactions on Medical Imaging 27, 161-170 (2008).

5 Fischl, B., Sereno, M. I., Tootell, R. B. H., and Dale, A. M. High-resolution inter-subject averaging and a coordinate system for the cortical surface. Human brain mapping 8, 272-284 (1999).

6 Hagler, D. J., Saygin, A. P., Stereno, M. I. Smoothing and cluster thresholding for cortical surface-based group analysis of fMRI data. Neuroimage 33 1093-1103 (2006).

**Functional MRI data acquisition and processing**

N-back working memory task

The N-back task parametrically increases the demands placed on working memory (WM), in this case over three levels (0-back, 1-back and 2-back). It was implemented as a blocked design, with letter stimuli being presented every 3s for 1s each, 14 per block. Blocks were preceded by a 7.5s instruction period, which informed the participant if this was to be a 0-, 1- or 2-back period. Total block duration was 49.5s. There were 3 repetitions of each level. Participants were asked to press button A if they saw a target letter, and B for any other. In the 0-back condition, the target was simply the letter X. For 1-back, the target was any letter that was the same as the immediately preceding one, and for 2-back the target was any letter that was the same as the one before that.

Data acquisition and analysis

Functional data were acquired using blood oxygen level-dependent echo-planar imaging (TR=1.56, TE=26ms, with 26 interleaved 4mm slices being acquired, separated by a 1mm gap, FOV=220mm, matrix= 64 x 64, in-plane resolution 3.44 x 3.44mm). 293 volumes were acquired over 457s, with the first 6 being discarded to avoid T1 saturation effects.

Behavioural analysis compared reaction times (RT) and sensitivity index, *d’*values, (calculated as Z(hit-rate) - Z(false-alarm-rate)) within a repeated measures design, with WM load as the within-group factor, covarying for age.

Functional data were preprocessed and analysed using SPM8 (http://www.fil.ion.ucl.ac.uk/spm/software/spm8/). First, slice-time correction to the 13th slice was performed. Data were then realigned to the mean EPI image, and volumes demonstrating >1.5mm of motion relative to the previous one were corrected by interpolating the immediately adjacent volumes. These volumes were encoded as nuisances within the first-level general linear model (GLM). The structural image was segmented, and MNI normalisation parameters applied to the EPI data following coregistration with the mean. Finally, data were smoothed using an 8mm FWHM gaussian kernel.

Within the first-level GLM, N-back blocks were encoded as boxcar functions, with each level of increasing demand being represented by a separate regressor. Realignment parameters were also included as covariates of no interest. The first-level T contrasts of 0-back > baseline, 1-back > baseline and 2-back > baseline were entered into a second-level factorial analyses modelling the effects of group and WM load. Both the main effects and the group X WM load interaction were examined. Activation maps were thresholded at *P*<0.005 uncorrected, and cluster correction for multiple comparisons applied at a family-wise level of *P*<0.05.

**Magnetic Resonance Spectroscopy data acquisition and analysis**

MRS protocol.

We acquired MRS point-resolved selective spectroscopy (PRESS) spectra with voxel placements in the ACC, right and left DLPFC. The voxels were shimmed using the Siemens advance mode. We acquired a water unsuppressed spectra with 16 averages and a water suppressed spectra with 128 averages. The TE was set to 80ms and the TR was set to 3000ms. The phase cycling was set to the Siemens 16 EXOR-cycle mode and the bandwidth was 2500Hz with over-sampling enabled. The voxels in the DLPFC measure 20 x 20 x 20 mm (8 cm^3^) and in the ACC measures 30 x 20 x 15 mm (9 cm^3^).

This followed a standardised protocol, with navigation steps to determine the coronal slice for placement. Cortical feature identification was used to designate the voxel centre, followed by rotations of the voxel in the transverse and sagittal views to obtain the final placement.

The location of the ACC voxel is shown in Figure 1. The coronal slice for voxel placement was found by moving 15 mm posterior from the genu of the corpus callosum (CC). In this coronal view the centre of the voxel was placed on the mid-line of the inter-hemisphere fissure, and 15mm above the dorsal surface of the CC. In the transverse view the voxel was rotated so that the anterior-posterior (long) axis followed the midline of the inter-hemisphere fissure. In the sagittal slice, the voxel anterior surface was aligned with the anterior edge of the genu of the CC. The voxel long axis was then rotated to follow the contour of the CC.


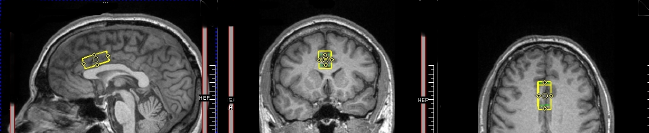
Figure 1. Voxel placement for MRS of the anterior cingulate cortex

The placement of the left DLPFC voxel is shown in Figure 2. The Talairach coordinates of the centre of the DLPFC voxel were x = +/-26, y = +24, z = +34. The coronal slice for voxel placement was found by moving 10 mm posterior from the genu of the corpus callosum. Then the voxel was centred in the white matter bordering the grey matter of the middle frontal gyrus (MFG). The transverse and sagittal slices were set by repeating the centering of the voxel in the white matter bordering the grey matter of the MFG. The voxel was then rotated to follow the contour of the cortex in the sagittal plane.


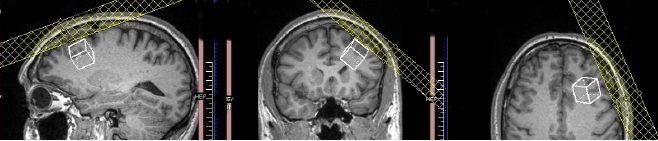
Figure 2. Voxel placement for MRS of the left dorsolateral prefrontal cortex

**Metabolite Measurement.** Spectral metabolite quantification was performed in LCModel version 6.2 (http://www.s-provencher.com/pages/lcmodel.shtml) using a gamma_press_te80_123mhz basis set provided at [http://s-provencher-com](http://s-provencher-com/) for use with the Siemens 3T Verio scanner. LCModel obtains maximum-likelihood estimates of metabolite concentrations and their uncertainties (Cramer-Rao lower bounds; CRLB). The raw spectra are read into the graphical user interface for LCModel. The spectroscopy data was processed through LC Model with eddy current correction enabled and internal water reference was used to give the metabolite in institutional units. For each voxel the metabolite values derived from LCModel were corrected for voxel cerebrospinal fluid (CSF) content as per the equation:

Met_CI_ = Met_I_ * (1 / 1 - F_CSF_)

Where *Met_CI_* is the metabolite in institutional units and corrected for partial volume effects, *Met_I_* is the internal water scaled metabolite value given by LC model and *F_CSF_* is the fractional CSF occupancy of the voxel. The fractional CSF volume was determined from the segmentation of the T_1_-weighted scan and the voxel placement and rotation noted at scan time. The T1-weighted scan was segmented into grey matter, white matter and CSF maps using SPM8 (Statistical Parametric Mapping; http://fil.ion.ucl.ac.uk/spm/). The CSF volume in each voxel was extracted using a c-script that sampled the SPM segmentation maps at the native space location noted for each voxel at time of scan.

**Analysis.** Initial reproducibility studies of both voxel placements and NAA/glutamate concentration values in the bilateral DLPFC and AC demonstrated excellent reliability (data available upon request). The quality of the spectra obtained and the specificity of the measurement of a given metabolite were evaluated using the percentage standard deviation. This is a measure of the specificity of the peak in the spectrum associated with a given metabolite. Only metabolite measurements that were associated with a CRLB of <30% were included in the analysis (up to 12 t(1;11) carriers, 16 non-carriers; but the N varies in each voxel). Analysis of covariance (ANCOVA) was preformed to examine the hypothesised differences in glutamate and NAA concentrations in each region in t(1,11) translocation carriers versus non-carriers individuals. Age and sex were entered in as covariates for all comparisons.

**Supplementary Results**

**Structural MRI results**

Diagnostic effects

For the brain regions where there were significant CT and LGI differences between translocation carriers and non-carriers, we plotted the data to further examine whether there were differences between those with a diagnosis of psychosis (PSY), those with recurrent depression (MDD) or those with other diagnoses (OTHERS) (Supplementary Results).

In the left hemisphere superior temporal sulcus region (Supplementary Figure 1 left), the cortical thickness values of all but one subject in the carrier group are smaller than the median value of the non-carrier group, and all sub-groups of translocation carriers have similarly lower thickness values than non-carriers. In the right hemisphere superior frontal region, the LGI values of all t(1;11) carriers were less than the median value of the non-carriers, see Supplementary Figure 1 (right).


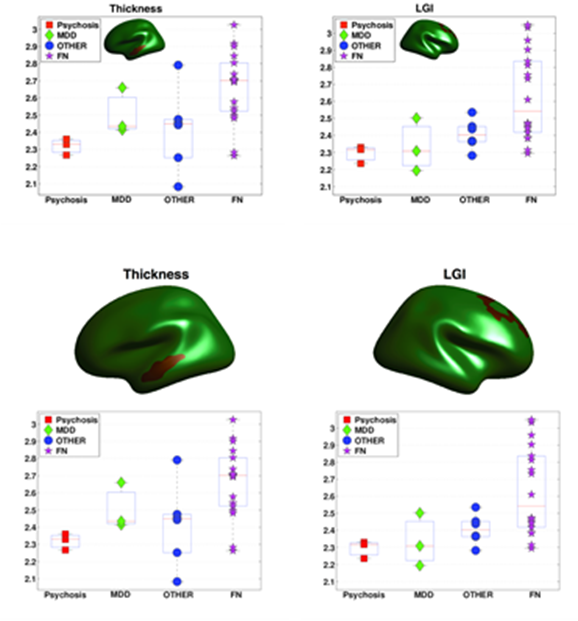


Supplementary Figure 1: The distribution of the cortical thickness and LGI value at the regions in which translocation carriers and non-carriers differ is highlighted on the brain surface. Individual values are presented for the t(1;11) carriers with SCZ, SCZAFF or BP (PSY), with recurrent depression (MDD), with other diagnoses (OTHER), and for non-carriers (FN). For each box plot, the central mark is the median, the edges of the box are the 25th and 75th percentiles, while the whiskers extend to the most extreme data points not considered outliers, and outliers are plotted individually.

1. Schaer M, Cuadra MB, Tamarit L, Lazeyras F, Eliez S, Thiran JP (2008): A surface-based approach to quantify local cortical gyrification*. IEEE transactions on medical imagi*ng. 27:161-170.
