# Supplementary material for: Balanced translocation linked to psychiatric disorder, glutamate, and cortical structure/function
Source: NPJ Schizophr. 2016 Aug 10;2:16024–. doi: 10.1038/npjschz.2016.24 (PMC4994153; doi:10.1038/npjschz.2016.24)
Supplement: Supplementary Dataset 1 [file npjschz201624-s2.doc]

Supplementary Dataset 1, 1a, 1b and 1c files have been removed as they contain patient confidential information.
